# Quantitative analysis in COVID-19: report of an initial experience

**DOI:** 10.31744/einstein_journal/2020AI5842

**Published:** 2020-10-02

**Authors:** Fábio Augusto Ribeiro Dalprá, Eduardo Kaiser Ururahy Nunes Fonseca, Gilberto Szarf, Rodrigo Caruso Chate

**Affiliations:** 1 Hospital Israelita Albert Einstein São PauloSP Brazil Hospital Israelita Albert Einstein, São Paulo, SP, Brazil.

A 63-year-old man came to the emergency room complaining of unverified fever and myalgia. Oropharyngeal material was collected for reverse transcription testing followed by polymerase chain reaction (RT-PCR) for coronavirus disease 2019 (COVID-19), and a chest radiography was performed (normal), and the patient was discharged to home isolation, due to his mild symptoms, until the test result.

After 3 days, the patient evolved with dyspnea, a drop in oxygen saturation (95%), and measured fever (38.6°C), and a chest computed tomography was performed (Figure 1) and he was admitted to hospital. The RT-PCR test for COVID-19 was positive.

Three days later, his respiratory pattern worsened, with a decrease in oxygen saturation to 90%, and he was referred to a stepdown unit and a new tomography was performed (Figure 1).

An 80-row CT scanner (Aquillion Prime, Canon Medical Systems, Tochigi, Japan) was used, with the patient in supine position, during maximum inspiration, and without injection of contrast medium. The following parameters were used: reconstructions with slice-thickness of 1 mm, a tube voltage of 80 kVp to 120 kVp, and adjustable current, varying between 10 mA and 440 mA. The images of these two exams were then processed, using the 3DSlicer software to segment the normal parenchyma, ground-glass opacities and consolidation areas in both lungs in the two exams performed for the patient (Figure 1).

From this, a quantitative analysis was conducted, showing in the first study a total lung volume of 4,289.62cm^3^, with the right lung measuring 2,214.91cm^3^ and the left lung measuring 2,074.71cm^3^. The preserved parenchyma area measured 2,110.48cm^3^ (95.29%) in the right lung, and 2,056.79cm^3^ (99.14%) in the left lung. The right lung presented with 81.01cm^3^ (3.66%) of ground-glass opacities and 23.42cm^3^ (1.06%) of consolidations; the left lung presented with 14.98cm^3^ (0.72%) of ground-glass opacities, and 2.95cm^3^ (0.14%) of consolidations. In total, the patient had 2.85% of parenchyma affected by ground-glass opacities or consolidations in the first study.

In the second study, the total lung volume calculated was 3,569.85cm^3^, with 1,814.95cm^3^ in the right lung and 1,754.90cm^3^ in the left lung. The preserved parenchyma area measured 915.17cm^3^ (50.42%) in the right lung, and 1,301.17cm^3^ (74.15%) in the left lung. The ground-glass opacity areas totaled 857.49cm^3^ (47.25%) in the right lung, and 447.63cm^3^ (25.51%) in the left lung, and the consolidation areas totaled 42.29cm^3^ (2.33%) in the right lung, and 6.09cm^3^ (0.35%) in the left lung.

In three days of progression, the patient showed an increase of 1,259.93% in ground-glass opacity volume, with 958.47% in the right lung, and 2,888.06% in the left lung, and an 83.44% increase in the consolidation volume, with 80.57% in the right lung, and 106.66% in the left lung. These numbers resulted in a 46.81% reduction in the preserved parenchyma volume, with a reduction of 56.64% in the right lung, and 36.74% in the left lung.

## DISCUSSION

Several cases of pneumonia of unknown origin that occurred in Wuhan, China, in late 2019, led to the discovery of a new type of coronavirus (2019-nCoV), called novel coronavirus-infected pneumonia (COVID-19).^(1-4)^ The virus quickly spread and started to affect individuals outside the initial contagion area, in other countries and, finally, on all continents, and was declared a pandemic by the World Health Organization (WHO).^(1-4)^

The 3DSlicer software is a free tool available online for download, whose use in quantitative imaging is well-established, having even been used in the evaluation of pulmonary nodules in chest imaging.^(5-7)^

Much has been studied since the beginning of the pandemic about the role of imaging tests in the prognosis and progressive control of COVID-19 patients, but a forceful answer has yet to be found. Our service, for instance, has been using the assessment of the tomographic progression of the disease as an auxiliary criterion in the clinical decision of hospitalization. The present case demonstrates the use of the 3DSlicer tool for the quantification of pulmonary tomographic changes, applied in the clinical monitoring of the patient, enabling an objective estimation of the involvement percentage and the progression rate of the disease. We believe that this tool can be an important resource for borderline cases or those that raise doubts about the significance of the progression. In addition, its association with artificial intelligence strategies can optimize the quantification process, rendering it possible the use of this quantification in a greater number of cases.

**Figure 1:**
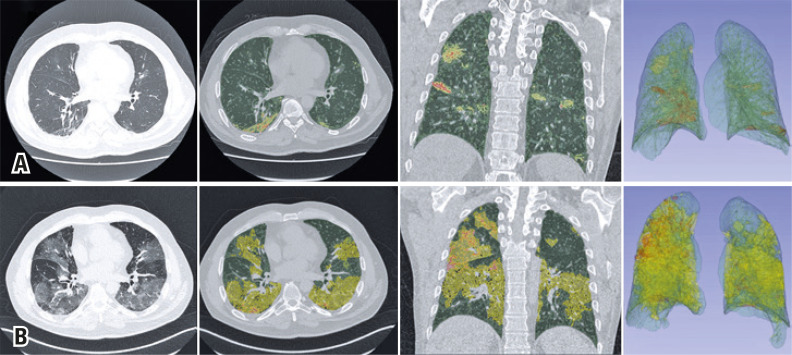
Chest computed tomography and superimposed 3DSlicer software quantification images. The upper series (A) show the findings when the patient returned to the emergency room, and the lower series (B) show the findings at the time of his clinical worsening. Axial sections of the chest tomography showing multifocal pulmonary ground-glass opacities predominantly peripheral and basal, more extensive in the last study, and quantitative images generated by the 3DSlicer software superimposed over the tomographic images. The areas marked in yellow show ground-glass opacities, those marked in green are areas of normal parenchyma, and those marked in orange are areas of consolidation. The extensive progression of the findings illustrates the numerical data provided
