# Supplementary material for: Quantitative analysis in COVID-19: report of an initial experience
Source: Einstein (Sao Paulo). 2020 Oct 2;18:eAI5842. doi: 10.31744/einstein_journal/2020AI5842 (PMC7531899; doi:10.31744/einstein_journal/2020AI5842)
Supplement: Translation

# Translation: Análise quantitativa na COVID-19: relato de uma experiência inicial

**Authors:** Fábio Augusto Ribeiro Dalprá, Eduardo Kaiser Ururahy Nunes Fonseca, Gilberto Szarf, Rodrigo Caruso Chate

**Affiliations:** 1 Brasil Hospital Israelita Albert Einstein, São Paulo, SP, Brasil.

Homem de 63 anos veio ao pronto atendimento com queixa de febre não aferida e mialgia. Realizou coleta de material de orofaringe para teste de transcrição reversa seguida de reação em cadeia da polimerase (RT-PCR) para a doença pelo coronavírus 2019 (COVID-19) e radiografia do tórax (normal), sendo encaminhado para isolamento domiciliar pelos sintomas brandos até o resultado do teste.

Evoluiu 3 dias depois com surgimento de dispneia, queda de saturação de oxigênio (95%) e febre aferida (38,6°C), sendo optada pela realização da tomografia computadorizada do tórax (Figura 1) e internação. Checado resultado positivo da RT-PCR para COVID-19.

**Figura 1:** Imagens de tomografia computadorizada do tórax e do programa de quantificação 3DSlicers sobrepostas. A linha superior (A) demonstra os achados no retorno do paciente ao pronto atendimento e a linha inferior (B) na ocasião de sua piora clínica. Cortes axiais da tomografia do tórax demonstrando opacidades pulmonares multifocais em vidro fosco de predomínio periférico e basal, mais extensas no último estudo, e imagens quantitativas sobrepostas às imagens tomográficas geradas pelo software 3DSlicer. As áreas marcadas em amarelo demonstram opacidades em vidro fosco, as em verde as áreas de parênquima normal e as em laranja as áreas de consolidação. Notar a extensa progressão dos achados, ilustrando os dados numéricos fornecidos

Três dias após, evoluiu com piora do padrão respiratório, com queda da saturação para 90%, sendo encaminhado para unidade semi-intensiva e realizada nova tomografia (Figura 1).

As imagens foram realizadas em equipamentos de 80 detectores (Aquillion Prime, Canon Medical Systems, Tochigi, Japão) com o paciente em decúbito dorsal, durante inspiração máxima e sem injeção do meio de contraste. Foram utilizados os seguintes parâmetros: reconstruções com 1mm de espessura, voltagem de 80 kVp a 120 kVp e corrente regulável, variando entre 10 mA e 440 mA. As imagens desses dois exames foram então processadas, utilizando-se o software 3DSlicer com o intuito de segmentar o parênquima normal, as opacidades em vidro fosco e as áreas de consolidação em ambos os pulmões nos dois exames realizados para o paciente (Figura 1).

A partir disto, foi possível realizar a análise quantitativa, que demonstrou um volume pulmonar total no primeiro estudo de 4.289,62cm^3^, sendo que o pulmão direito possuía 2.214,91cm^3^ e o esquerdo 2.074,71cm^3^. A área de parênquima preservado de cada pulmão era de 2.110,48cm^3^ (95,29%) à direita e 2.056,79cm^3^ (99,14%) à esquerda. O pulmão direito apresentou 81,01cm^3^ (3,66%) de opacidades em vidro fosco e 23,42cm^3^ (1,06%) de consolidação; já o esquerdo apresentou 14,98cm^3^ (0,72%) de opacidades em vidro fosco e 2,95cm^3^ (0,14%) de consolidação. No total, o paciente apresentou 2,85% de parênquima acometido por vidro fosco ou consolidações no primeiro estudo.

Em seu segundo estudo, o volume pulmonar total calculado foi de 3.569,85cm^3^, sendo 1.814,95cm^3^ no pulmão direito e 1.754,90cm^3^ no pulmão esquerdo. A área de parênquima preservado foi de 915,17cm^3^ (50,42%) à direita e 1.301,17cm^3^ (74,15%) à esquerda. As áreas de vidro fosco somaram 857,49cm^3^ (47,25%) à direita e 447,63cm^3^ (25,51%) à esquerda, e as áreas de consolidação somaram 42,29cm^3^ (2,33%) à direita e 6,09cm^3^ (0,35%) à esquerda.

Em 3 dias de evolução, o paciente apresentou aumento de 1.259,93% no volume de vidro fosco, sendo de 958,47% à direita e 2.888,06% à esquerda, e aumento de 83,44% no volume de consolidação, sendo 80,57% à direita e 106,66% à esquerda. Tais números se traduziram numa redução de 46,81% de volume de parênquima preservado, sendo essa redução de 56,64% à direita e 36,74% à esquerda.

## DISCUSSÃO

Diversos casos de pneumonia de origem desconhecida ocorridos no final de 2019 em Wuhan, China, levaram à descoberta de um novo tipo de coronavírus (2019-nCoV), denominado *novel coronavirus-infected pneumonia* (COVID-19).^(1-4)^ Esse vírus rapidamente se disseminou e passou a afetar indivíduos fora da área inicial de contágio, em outros países e, por fim, em todos os continentes, ganhando estado de pandemia pela Organização Mundial da Saúde (OMS).^(1-4)^

O software 3DSlicer é uma ferramenta gratuita e disponível online para download, que tem seu uso bem estabelecido em imagem quantitativa, inclusive já tendo sido utilizado em imagem torácica para, por exemplo, avaliação de nódulos pulmonares.^(5-7)^

Muito tem sido estudado desde o início da pandemia sobre o papel dos exames de imagem no prognóstico e no controle evolutivo desses pacientes, ainda sem ter sido encontrada uma resposta contundente. Nosso serviço, por exemplo, vem usando a avaliação da extensão tomográfica da doença como critério auxiliar na decisão clínica de internação. O presente caso demonstra a utilização da ferramenta 3DSlicer para a quantificação das alterações tomográficas pulmonares, aplicada no controle evolutivo, permitindo estimar, de forma objetiva, o percentual de acometimento e a taxa de evolução. Acreditamos que essa ferramenta possa ser apoio importante para casos limítrofes ou que suscitam dúvidas quanto à significância da progressão. Além disso, sua associação com estratégias de inteligência artificial pode otimizar o processo de quantificação, fazendo com que essa quantificação possa ser adotada em um maior número de casos.
